# From Shadows to Spotlight: Enhancing Bacterial DNA Detection in Blood Samples through Cutting-Edge Molecular Pre-Amplification

**DOI:** 10.3390/antibiotics13020161

**Published:** 2024-02-06

**Authors:** Martin Reinicke, Sascha Daniel Braun, Celia Diezel, Oliver Lemuth, Ines Engelmann, Theresa Liebe, Ralf Ehricht

**Affiliations:** 1 Leibniz Institute of Photonic Technology (IPHT), Leibniz Centre for Photonics in Infection Research (LPI), 07745 Jena, Germany; 2 InfectoGnostics Research Campus, 07743 Jena, Germany; 3 BLINK AG, 07747 Jena, Germany; 4 Institute of Physical Chemistry, Friedrich-Schiller University, 07743 Jena, Germany

**Keywords:** sepsis, blood culture, pre-amplification, PCR, multiplex analysis, pathogen diagnosis, AMR, sample preparation

## Abstract

One of the greatest challenges to the use of molecular methods for diagnostic purposes is the detection of target DNA that is present only in low concentrations. One major factor that negatively impacts accuracy, diagnostic sensitivity, and specificity is the sample matrix, which hinders the attainment of the required detection limit due to the presence of residual background DNA. To address this issue, various methods have been developed to enhance sensitivity through targeted pre-amplification of marker sequences. Diagnostic sensitivity to the single molecular level is critical, particularly when identifying bloodstream infections. In cases of clinically manifest sepsis, the concentration of bacteria in the blood may reach as low as one bacterial cell/CFU per mL of blood. Therefore, it is crucial to achieve the highest level of sensitivity for accurate detection. In the present study, we have established a method that fills the analytical gap between low concentrations of molecular markers and the minimum requirements for molecular testing. For this purpose, a sample preparation of whole blood samples with a directly downstream pre-amplification was developed, which amplifies specific species and resistance markers in a multiplex procedure. When applying pre-amplification techniques, the sensitivity of the pathogen detection in whole blood samples was up to 100 times higher than in non-pre-amplified samples. The method was tested with blood samples that were spiked with several Gram-positive and Gram-negative bacterial pathogens. By applying this method to artificial spiked blood samples, it was possible to demonstrate a sensitivity of 1 colony-forming unit (CFU) per millilitre of blood for *S. aureus* and *E. faecium*. A detection limit of 28 and 383 CFU per ml of blood was achieved for *E. coli* and *K. pneumoniae*, respectively. If the sensitivity is also confirmed for real clinical blood samples from septic patients, the novel technique can be used for pathogen detection without cultivation, which might help to accelerate diagnostics and, thus, to decrease sepsis mortality rates.

## 1. Introduction

Despite advancements in medical technology and healthcare systems, the global mortality rate associated with bloodstream infections remains alarmingly high [1]. Classical methods for diagnosing bacteraemia and/or sepsis involve detecting bacterial growth in liquid media inoculated with patient blood, i.e., blood cultures, followed by pathogen identification and susceptibility testing. The incubation of the blood culture usually requires a period of 24 to 72 h [2]. Additionally, slightly more than half of patients clinically diagnosed with sepsis or septic shock had a positive blood culture [3,4]. Until an identification and a susceptibility test become available, therapy relies on a calculated administration of antibiotics that is based on the clinical presentation, on the presumed focus of the infection, on knowledge about local outbreak strains, and on local statistics regarding antibiotic resistance. This poses a risk of therapy failure in individual patients, and this dilemma might also result in an over-prescription of broad-spectrum and last-line antibiotics (such as carbapenems) resulting in an increased selective pressure favouring multiresistant bacteria [5,6].

Molecular methods in clinical diagnostics offer clear advantages in terms of speed over conventional approaches, especially for “hard-to-cultivate” or slow-growing microbes [7,8]. For molecular methods without prior enrichment by incubation, a bottleneck is caused by the sample matrix effects of blood, pus, or faeces and mainly by the low bacterial loads of causative pathogens in the sample [9,10]. Whole blood samples contain various components, such as haemoglobin [11], immunoglobulins [12], and anticoagulants, like heparin [13,14], which can inhibit downstream PCR. The high concentration of human DNA leads to an inhibitory effect through non-specific binding of primers and probes, resulting in loss of specificity and performance [15].

In this context, both the sample preparation to isolate the target DNA and the selective amplification of molecular markers are crucial for the sensitivity and specificity of molecular-based diagnostics. The challenge is to multiply the target sequences above a threshold to detect them with downstream diagnostic methods, like qPCR, DNA microarrays, or lateral flow assay combined with molecular isothermal amplification methods [16,17,18,19].

An additional challenge emerges when aiming to detect one or multiple molecular targets with both high sensitivity and specificity in a single PCR reaction, without prior knowledge of the target composition or copy numbers in the sample. Such reactions often run as at least a duplex reaction (i.e., with one target plus an internal process control, IPC) or commonly as a multiplex reaction (with multiple targets, such as a species marker plus some important resistance genes plus an IPC). All these reactions compete for the same resources, like oligonucleotide primers, dNTPs, magnesium, and polymerase, and this competition might result in false-negative results for reactions that start with a lower target number or perform less efficiently for other reasons. Therefore, achieving accurate multiplex amplifications can be challenging, especially when dealing with varying initial concentrations of target DNA. One of the main challenges of multiplex amplification is to ensure that all target sequences are amplified with equally or comparably high sensitivity and specificity, regardless of their initial concentration.

Therefore, the goal of our study was to achieve equal concentrations for all intended targets in hard-to-analyse whole blood samples in order to facilitate reliable and robust results of subsequent molecular assay procedures, such as qPCR. For this achievement, a new pre-amplification process was developed, and our primary objective was to amplify all target DNA molecules to a threshold of 10^3^ genomic equivalents (GE) per reaction. We also addressed the fundamental issue of DNA isolation by developing a simple method without the need for specific laboratory equipment to prepare and enrich bacterial nucleic acids from whole blood samples with low bacterial loads. This involved a cascade of different filtration steps followed by a selective and highly sensitive amplification procedure in a multiplex approach for several genetic markers. Combining both processes, i.e., whole blood DNA isolation and pre-amplification, could be a future solution for molecular assays in sepsis diagnostics, achieving sensitivity and specificity without the need for blood culture.

## 2. Results

### 2.1. Pre-Amplification Procedure

The pre-amplification method described herein was adapted from the round A/B technology for sequence-independent amplification previously described by Bohlander and colleagues [20].

Our adapted method was based on target-specific primer binding of chimeric oligonucleotides (primer A) followed by a parallel and universal amplification for all targets implemented by a secondary unique primer (primer B). Primer A consisted of two regions, namely a target-specific sequence and the complementary sequence as a binding site for the secondary primer B. Primer B was an artificial sequence that yielded no complete hit in the NCBI nucleotide database for the panel of bacterial species (last check November 2023).

**Figure 1:**
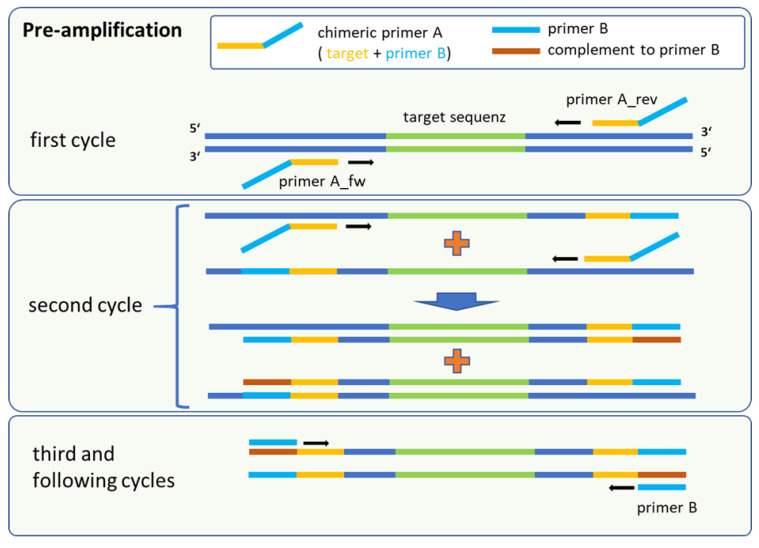
Schematic procedure of the pre-amplification method, which is a sequential amplification followed by universal marker gene multiplication. The fusion primer A consisted of a target-specific part (yellow) and the complementary sequence of primer B. Binding sites for primer B (red) were first synthesised in the second cycle.

In the initial steps, only primer A is active for the target-specific amplification and incorporation of binding sites for a subsequent universal multiplication of marker genes (Figure 1). Following the denaturation step in the first cycle, the target-specific sequence of primer A hybridises with the single-stranded DNA in a sequence-specific manner. Annealing and elongation were performed in one step at 43 °C for 15 s, releasing double stranded fragments with the terminal sequence for primer B at the 5′ end of the amplicon. This intermediate amplification product served as template after denaturation in the next stage. Binding of the corresponding forward or reverse version of primer A led to the synthesis of fragments with the complementary sequence for primer B. At this point, the amplification is driven mainly by primer B, because the amplification was uncoupled from the specific binding of primer A to the marker sequence. In parallel, forward and reverse primer A generated still more target fragments of marker genes flanked by the binding sites of primer B. The reaction took place in one mix and allows a high amplification rate for different targets sequences in a multiplex format. For quantification of the pre-amplification rates, marker-specific monoplex qPCR assays were used to compare the output with the input of the reaction. The calibration curves for each primer/probe set in the monoplex qPCR assay were performed using 20 genomic equivalents (GE) per reaction as the lowest standard for all targets (Table A1). This was the lowest dilution that consistently produced Ct values within the linear range. Serially diluted genomic DNA was utilised as a template. In cross-hybridization experiments that involved all genomic DNAs in combination with each primer pair for all markers, no false-positive results were detected (Table A2 and Table A3). All primer/probe sets showed a high sensitivity and specificity for their respective targets.

**Figure 2:**
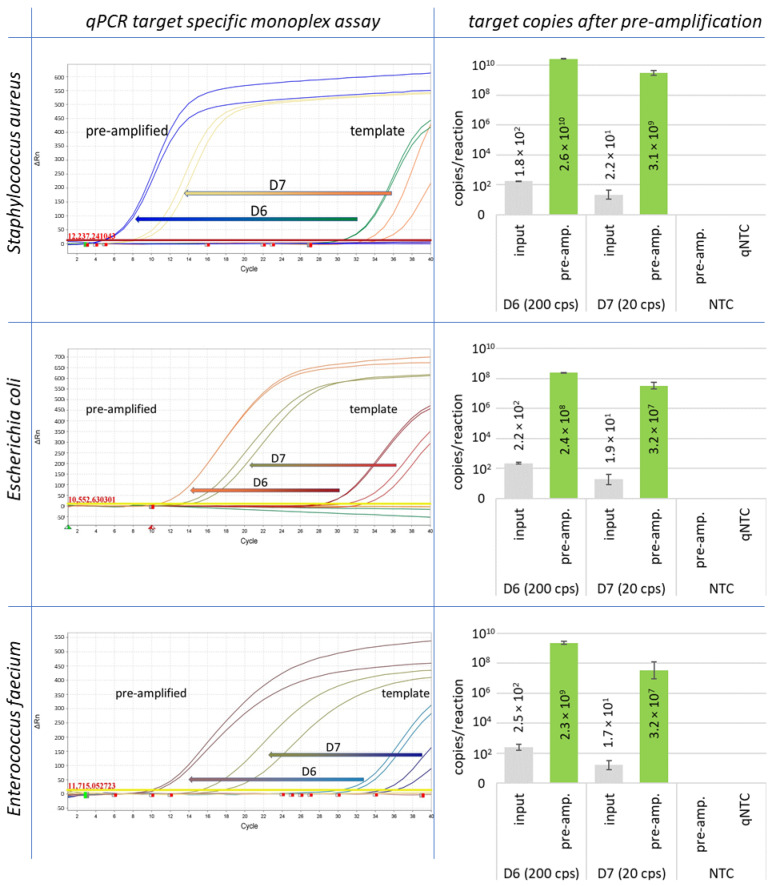
Pre-amplification results are shown for *Staphylococcus aureus*, *Escherichia coli*, and *Enterococcus faecium*. The starting concentration was 200 GE (D6) and 20 GE (D7) of genomic DNA per reaction.

For the development of the method, the pre-amplification reaction was initially carried out only with genomic DNA of all different reference strains in 20 µL volumes with 200 and 20 GE per reaction as template. All markers have been successfully amplified to at least 10^4^ copies per reaction mix with an initial number of 20 GE per reaction (Table 1, column IPC). Monoplex assays and resulting target copy numbers for pre-amplification experiments with *Staphylococcus aureus*, *Escherichia coli*, and *Enterococcus faecium* are shown in Figure 2.

Human DNA was used as the background in the pre-amplification reactions to simulate the sample matrix effects. It was calculated that after preparation of a 3 mL blood sample, approximately 800 ng of human DNA remained in the sample. The proportion of residual human DNA was estimated based on the efficiency of filtration steps in the sample preparation. The calculation was based on the remaining cell numbers and the average content of human DNA. Thus, a total amount of 1 µg human DNA was added to each sample as an artificial sample matrix in order to test the stability and robustness of the method. The reaction was performed as previously described, and a 2 µL aliquot was used as a template and analysed by qPCR.

**Figure 3:**
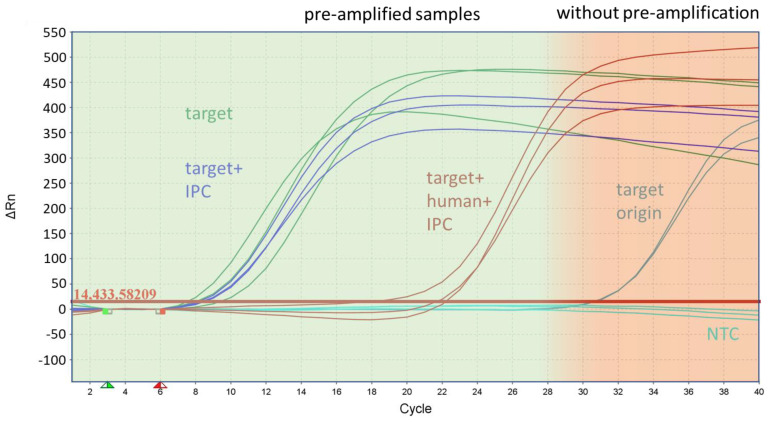
Inhibitory effect of human DNA during pre-amplification on the target amplification rate of the species marker *basC* for *Acinetobacter baumannii*. The figure shows the curves of the monoplex qPCR assay from samples with the target origin (200 GE/reaction), which was the starting concentration of the samples in front of the pre-amplification, and the pre-amplified samples after with the target and with the IPC and target, using human DNA and the IPC as the template.

The efficiency of amplification was affected by the high amount of human DNA, and the number of amplified target DNA was reduced in a range of two or three orders of magnitude. However, despite the presence of a high background of human DNA, all markers were successfully amplified above a threshold of 10^3^ genome equivalents (GE) per reaction for downstream applications, as demonstrated in Figure 3. for *basC* (*Acinetobacter baumannii*). Each specific marker for the individual bacterial strains exhibited significant amplification and multiplication over several orders of magnitude. The resulting final concentration of genome equivalents for each individual species and resistance marker after pre-amplification was between 10^5^ and 10^8^ GE/reaction, using an initial template concentration of 20 GE of bacterial DNA per reaction without human DNA (Table 1). For most marker genes, pre-amplification resulted in a concentration ranging from 10^6^ to 10^7^ GE/reaction.

### 2.2. Multitarget Pre-Amplification

**Figure 4:**
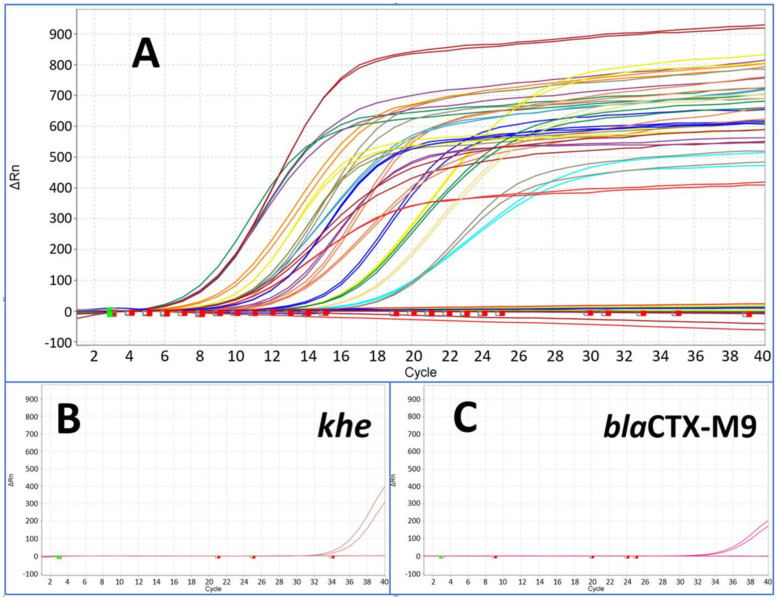
The figure shows results from pre-amplified samples after pre-amplification followed by downstream monoplex qPCR assay for all species and resistance marker (**A**). The starting point was a template concentration of 200 GE per reaction of each marker with Ct values from around 32 up to 34. All markers showed Ct values between 6 and 18 after pre-amplification (compared with Figure 3) with the exception of *khe* (**B**) and *bla*CTX-M9 (**C**) at around Ct 34.

In the context of analysing bacterial infections, we investigated the feasibility of simultaneously amplifying all 22 target genes (including species identifiers and resistance markers) in a single pre-amplification reaction, whether these targets reside within the same bacterium or across different bacterial strains. This innovative approach allowed for the parallel pre-amplification of all targets within a single reaction mix. The downstream duplex qPCR assay (target and IPC) revealed a high efficiency for all markers with the exception of the markers *khe* (the species marker of *Klebsiella pneumoniae*) and *bla*CTX-M9 (ESBL) (see Figure 4).

### 2.3. Blood Sample Spiking Experiments

To verify the method with typical clinical samples with regard to sepsis and bacteraemia diagnostics, blood samples from healthy donors were spiked with defined amounts of *Escherichia coli*, *Klebsiella pneumoniae*, *Staphylococcus aureus*, and *Enterococcus faecium*. As internal process control, spores of *Bacillus atrophaeus* were immobilised in the first used syringe for sample preparation. For each strain a fresh culture was prepared, and bacteria were added at a theoretical final concentration of 10^3^, 10^2^, and 10^1^ CFU/mL blood. The accurate CFU numbers were determined by plate counts. A sample volume of 3 mL blood was prepared for DNA extraction. From the eluate (75 µL), an aliquot of 50 µL were used as template for pre-amplification and 2 µL was used as the template for comparison in qPCR.

**Figure 5:**
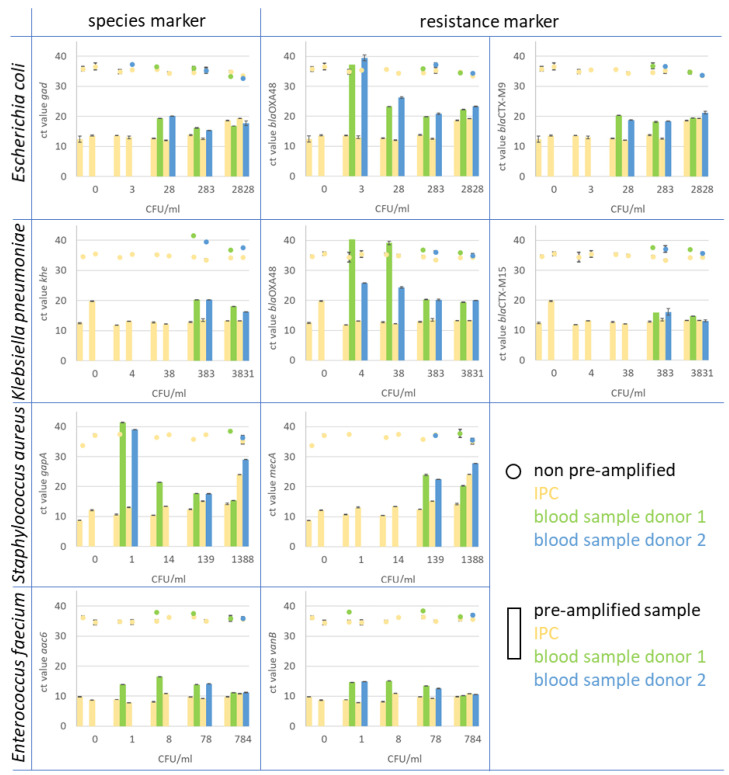
In the figure, the Ct values of monoplex qPCR assays for species and resistance markers are presented. The assays were performed on spiked whole blood samples after pre-amplification treatment and compared to spiked and prepared blood samples that were not pre-amplified. Blood samples from two healthy donors (blue and green) were spiked with *Escherichia coli*, *Klebsiella pneumoniae*, *Staphylococcus aureus*, and *Enterobacter faecium*. As IPC, spores of *B. atrophaeus* (yellow) were introduced at the beginning of the blood sample preparation. The pre-amplified samples (bars) showed a clear increase in target copy number in comparison to the original sample material (dots). For instance, the resistance marker CTX-M9 in *E. coli* (upper right diagram) was detected in spiked blood samples from both donors 1 and 2 (green bar for donor 1 and blue bar for donor 2) at a concentration of 28 CFU/mL blood after pre-amplification. The marker was detected with Ct values of 20.3 for donor 1 and 18.2 for donor 2. In non-pre-amplified samples (green dots for donor 1 and blue dot for donor 2), the marker was found in samples with 283 CFU/mL blood with Ct values of 36.8 and 36.7, respectively. The corresponding IPC to each blood sample is displayed in yellow for non-pre-amplified (dot) and pre-amplified samples (bar).

In general, the majority of pre-amplified samples consistently showed significantly earlier positive qPCR signals for the spiked bacteria compared to the non-amplified samples. Notably, the highest sensitivity for species markers in pre-amplified samples was observed for Enterococcus faecium, reaching a detection threshold as low as 1 CFU/mL (as illustrated in Figure 5). Similarly, *Staphylococcus aureus* was detected with the same sensitivity, at 1 CFU/mL, in the spiked blood sample. It is noteworthy that this heightened sensitivity was achieved in some instances but not consistently across all samples. Notably, the *gapA* monoplex assay yielded a high cycle threshold (Ct) value at this concentration for both blood donors. For *Escherichia coli* and *Klebsiella pneumoniae*, the sensitivity level using the pre-amplification method was 28 and 383 CFU/mL blood, respectively. For Gram-negative bacteria, including *Escherichia coli*, *Klebsiella pneumoniae*, and *Enterococcus faecium*, the various resistance markers were detected with equal or even higher sensitivity compared to the species markers; for example, the resistance marker (carbapenemase) b*la*OXA-48, in *E. coli* and *Klebsiella pneumoniae* strains, was detected in the blood sample with a LOD of 3 CFU/mL in the *E. coli* and with 4 CFU/mL in the *Klebsiella pneumoniae* sample. While in *E. coli* ID 240608, this gene is located on the chromosome, it is localised on a plasmid in *Klebsiella pneumoniae* ID 239644. The *bla*CTX-M15 and M9 genes, which encode for ESBL, were found on the chromosome in both strains. The genes were detected with the same sensitivity as the chromosomal species markers *gad* and *khe*. The resistance marker *mecA* in *Staphylococcus aureus* was found clearly earlier in samples spiked with a lower CFU count than the species marker *gapA* and was found first in the sample with 139 CFU/mL. The vancomycin resistance *vanB*, associated with *E. faecium*, was detected with the same sensitivity as the corresponding species marker *aac6.*

In summary, pre-amplification has demonstrated a substantial enhancement in detection sensitivity, even within spiked blood samples. This is especially significant in the context of diagnosing sepsis, as the number of bacteria present in the bloodstream is usually very low. Pre-amplification facilitates the early detection of pathogenic bacteria in real-world samples, such as blood, without the necessity for time-consuming cultivation methods. This approach optimises both sensitivity and specificity while enabling a multiplex format, ensuring a comprehensive and efficient molecular diagnostic solution.

## 3. Discussion

Full blood samples are not very favourable for the identification of bloodstream infections in direct analysis by molecular-based methods without any cultivation step before. The direct analysis from full blood samples has to deal with several limiting effects, like high human DNA background, PCR-inhibiting substances, low concentration of pathogen DNA, and high sample volumes for the needed sensitivity and free bacterial DNA in the blood. The aim of this study and the method described herein was to reduce the limiting and inhibiting effects and make such samples more attractive and usable for common downstream nucleic acid-based analyses. Both culture-dependent and molecular-based methods to diagnose bloodstream infections are constrained by limitations. The culture-dependent gold standard blood culture needs a significant amount of time and only provides information about the species or possible resistance of the causative micro-organisms with further diagnostics. Furthermore, the blood culture can stay negative due to prior therapeutic antibiotic treatment and the limited growth of the pathogens [21]. The benefits of molecular cultivation-independent techniques lie in their high speed, sensitivity, and specificity. This enables the rapid generation of information on species and resistance [22,23]. Nonetheless, it remains unclear whether the resistance genes detected are phenotypically expressed and, therefore, confer resistance. An analysis of currently available molecular test systems clearly shows that mostly only a few pathogens can be detected directly from whole blood. In many instances, the method is restricted in the detection of a small spectrum of pathogens or resistance markers with high sensitivity, or the variability of the method is limited for the addition of other markers (Table A4).

In addition to traditional amplification methods for detection, diagnostic applications are increasingly relying on next-generation sequencing (NGS). Sequencing provides very high specificity and, depending on the sample preparation, sensitivity. The limitations are primarily related to sample preparation, costs and effort, and processing the raw sequencing data [23,24].

### 3.1. Sample Preparation for Improved Sensitivity

In this study, our newly developed sample preparation method effectively minimised the influence of the sample matrix and background human DNA, resulting in enhanced target-specific DNA amplification. The sensitivity of the method is highly dependent on the filtration steps. Our experience has shown that filtration of up to 3 mL of blood is effective with the chosen filter dimensions and that the leukocytes can be easily removed from the sample. However, larger sample volumes can cause the filters to clog quickly, resulting in false-negative results by trapping the bacteria in the clots at the filters. Filtering leukocytes, lysing erythrocytes, and then filtering cell debris removes over 90% of human material, including DNA, which can negatively affect amplification. After crucially flushing the filters to remobilise bacteria from the filter surface or pores, the retrieved bacteria were subjected to downstream mechanical lysis and DNA isolation. This sequential separation of pathogens followed by their lysing significantly improved the performance of targeted DNA amplification for pathogenic bacteria. Additionally, cell-free bacterial DNA from dead bacterial cells, which circulates in the bloodstream and is not related to the infection, is also removed during the filtration and does not lead to false-positive results anymore [25]. On the other hand, the recovery rates for various pathogens when separated by filtration was mostly independent from the blood samples and their composition. In the spiked blood specimens for nearly all pathogens, comparable levels were reached for the different targets in comparison to the blood samples from different donors. For *Enterococcus faecium*, there was a significant disparity of nearly two orders of magnitude in the sensitivity of detecting the species marker *aac6* between the two blood samples obtained from different donors.

### 3.2. Advantages of the Pre-Amplification Method

The introduction of an artificial sequence in the pre-amplification step significantly improved the uniformity of amplification rates, reducing the need for complex primer concentration titration experiments. The separation of the workflow into semi-sequence-specific pre-amplification and sequence-specific quantitative monoplex amplification markedly increased specificity while minimising cross-reactions. The pre-amplification method exhibited robustness, stability, and high sensitivity, making it suitable for a wide range of targets (Table A2 and Table A3). The pre-amplification method is stable against the inhibitory effect of human DNA (up to 50 ng/µL) and produces target copy numbers that are two or more orders of magnitude above the established threshold of 10^3^ copies/reaction for all markers, which are required for further downstream applications. Using a threshold value of 10^3^ copies/reaction, it was deemed unnecessary to determine an exact limit of detection (LoD) for the monoplex assays during method development. The LoD for the pre-amplification method was determined to be between two sample points of the dilution series in the experiments with spiked blood samples. If there are intentions for further commercialisation of the method, a concrete determination of the LoD by using finely graded sample dilutions in the range of the detection limit and sufficient sample parallels would be necessary. The determination of the limit of detection (LoD) for a molecular DNA amplification diagnostic method is crucial for its commercialization.

Several methods and guidelines are available for determining the LoD. One approach is to use finely graded sample dilutions in the range of the detection limit and sufficient sample parallels. The limit of detection (LoD) and the limit of quantification (LoQ) can be determined using methods applicable to quantitative real-time PCR (qPCR). The LoD is usually determined by using a set of log-diluted controls, such as patient samples, a suitable cell line, or proficiency panels, with a positive call rate above 95% for the standard samples containing the target molecules. Additionally, a new method based on maximum likelihood estimation (MLE) of LoD using a theoretical math model of concentration has been described, which is free of the flaws of the method based on an empirical model. It is important to conduct the LoD evaluation study with practical considerations and to verify the LoD for the molecular diagnostic assay using a real-time polymerase chain reaction (PCR). These methods and guidelines provide a framework for conducting an approval study for the commercialization of a molecular DNA amplification diagnostic method [26,27,28].

Another benefit of this method is its simplicity in expanding the range of targets. This can be achieved by adding the semi-specific primers to the pre-amplification primer set and validating the corresponding monoplex assays using the multi-DNA pre-amplification as a template and testing for cross-reactions with already included and tested targets.

### 3.3. Considerations for Contamination and Closed Systems

The method’s high sensitivity made it prone to generating false-positive results through the introduction of foreign DNA. False-positive signals in PCR can arise due to several reasons; these include bacterial or free DNA contamination during sample processing, residual foreign DNA in PCR reaction compounds, and the detection of commensals introduced during sample collection.

In the present study, the sample preparation stage was identified as the most at-risk step for contamination due to the multiple liquid transfers involved. To prevent contamination, it is important to perform the workflow in a closed system, such as a cartridge. Therefore, it is recommended to use a closed system and ensure to DNA-free and sterilised components.

Traces of DNA, which may be present in PCR reagents produced using recombinant organisms, can result in positive signals for species-specific target markers related to the microorganisms of the expression systems. Single DNA molecules remaining in recombinant-produced amplification compounds, which are not observed in standard qPCR assays, resulted in verifiable and reproducible false-positive signals when using the pre-amplification method.

The inadvertent introduction of widespread microbial skin commensals, such as *Staphylococcus epidermidis*, during blood sample acquisition remains unresolved and can lead to potentially incorrect positive test results. However, it is difficult to distinguish between bacteria that cause bloodstream infections and those that are commensal within the same species without additional screening [29].

### 3.4. Clinical Relevance: Achieving a Critical Detection Level

We have developed a very robust, stable, and variable method to prepare and amplify traces of pathogenic DNA in human blood samples to a diagnostically detectable level for conventional molecular methods. Our combined sample preparation and pre-amplification method has successfully achieved a sensitivity level of 1 CFU/mL in blood, a critical threshold for defining sepsis. This is a significant advancement in the field, potentially allowing for the early diagnosis of sepsis without the need for time-consuming cultivation methods.

It is important to note that the blood samples used in this study were deliberately spiked with bacteria and, therefore, may not accurately reflect the nature of real clinical blood samples from septic patients. As a result, the behaviour of these samples during sample preparation may also differ.

With the method, a panel of molecular species and resistance markers was amplified in parallel. Nonetheless, pre-amplification is viable for clinical samples, such as blood or liquor, where the sample matrix meets low loads of pathogenic bacteria.

Improved diagnostics are crucial in addressing the issue of antibiotic resistance strategically. Prioritising faster and more accurate diagnostic results can decrease the use of empirical therapies and encourage the timely use of targeted drugs. The study’s findings align with the overarching objective of preventing and mitigating the emergence and impact of multi-drug resistant bacteria.

## 4. Materials and Methods

### 4.1. Species and Resistance Marker Panel

For the identification of microorganisms that cause bloodstream infections, a panel of species-specific markers and resistance genes was selected. This panel included 12 species markers for Gram-positive (n = 7) and Gram-negative (n = 5) bacteria, as well as 12 genes associated with resistance to carbapenems (n = 7), third-generation cephalosporins (n = 2), methicillin (n = 1), and vancomycin (n = 2) (Table 2). 

Genomic DNA of the *Bacillus atrophaeus* was used as the internal process control (IPC) for the pre-amplification, followed by the final monoplex qPCR assays.

### 4.2. Bioinformatics for Oligonucleotide Design

For each marker, multi-FASTA alignments were prepared using MAFFT (version v7.475). Chimeric oligonucleotides for pre-amplification (Table 3) as well as primers and TaqMan probes (Table 4) for monoplex qPCR assays were designed with ConsensusPrime [30]. The TaqMan-probes were labelled with different fluorophores (i.e., 6-Fam or ATTO647) covalently attached to the 5′ end. All oligonucleotides were synthesised by Metabion (Steinkirchen, Germany).

### 4.3. Bacterial Strains and Growth Conditions

In order to assess the efficacy of the method, various strains from our inhouse strain collection were utilised as reference. The panel consisted of 24 strains encompassing 10 different species, namely *Acinetobacter baumannii*, *Citrobacter freundii*, *Enterococcus faecalis*, *Enterococcus faecium*, *Escherichia coli*, *Klebsiella pneumoniae*, *Pseudomonas aeruginosa*, *Staphylococcus aureus*, *Staphylococcus epidermidis*, and *Streptococcus pneumoniae* (Table 5). All strains were cultivated on Columbia blood agar (Becton Dickinson, Heidelberg, Germany) overnight at 37 °C. All reference strains were additionally characterised by antibiotic susceptibility tests using a VITEK-2 system according to the manufacturer’s instructions and following the EUCAST guidelines (version 2023). Furthermore, all strains were subjected to ONT next-generation whole genome sequencing to evaluate allelic variants and to determine whether resistance genes were chromosomally encoded or plasmid-borne.

### 4.4. Nucleic Acid Preparation and Sequencing

DNA extraction for Nanopore MinION sequencing (Oxford Nanopore Technology, Oxford, UK) was carried out using a Nucleospin Microbial DNA Kit by Macherey Nagel (MN, Düren, Germany). Therefore, all strains were cultured from cryo-cultures (Microbank; ThermoFisher Scientific, Waltham, MA, USA) on blood agar plates at 37 °C overnight. One full inoculation loop per strain was then washed with 500 µL 1× PBS (pH 7.4), centrifuged, and resuspended in 100 µL buffer BE. All subsequent steps were conducted following the manufacturer’s instructions with two minor adaptations: (1) Samples were lysed using a bioshaker (QINSTRUMENTS, Jena, Germany) for 12 min (Gram-positive bacteria) or, respectively, 4 min (Gram-negative bacteria) at full speed. (2) Before binding the DNA onto Nucleospin microbial DNA columns, proteinase K was inactivated by incubating the samples at 70 °C for 5 min. After the sample cooled down, 4 µL of RNAse (100 mg/mL; Sigma Aldrich, Steinheim, Germany) was added and samples were incubated at 37 °C for 5 min. Finally, DNA was eluted twice with 75 µL of nuclease-free water (Carl Roth, Karlsruhe, Germany).

For genome sequencing of all 24 strains, 5 MinION flow cells and 7 flongle flow cells (R9.4.1) were used. Library preparations were performed using the 1D genomic DNA ligation kit (SQK-LSK 109) and the native barcoding expansion kits (EXP-NBD103, EXP-NBD104, and EXP-NBD114). In short, size selection and DNA clean-up were performed using Agencourt AMPure XP beads (Beckman Coulter GmbH, Krefeld, Germany) at a ratio of 1:1 (*v*:*v*) prior to library preparation. Potential nicks in DNA and DNA ends were repaired in a combined step using NEB Next FFPE DNA Repair Mix and the NEB Next Ultra II Ned repair/dA-tailing module (New England Biolabs. Ipswich, MA, USA) by tripling the incubation time. Prior to adapter ligation, barcodes were ligated to the dA-prepared ends of the DNA and a second AMPure clean-up was performed. A subsequent third AMPure bead purification was followed by the ligation of sequencing adapters onto prepared ends. At the start of sequencing, an initial quality check of each flow cell showed a minimum of 1200 active pores. Genomic DNA samples used for loading comprised a total amount of around 40 to 60 ng per strain (measured by Qubit 4 Fluorometer; ThermoFisher Scientific). The sequencing ran for 72 h using the MinKNOW software versions 22.05.5.

The guppy basecaller (version 4.5.2 + bcc53d392 up to 6.0.1 + 652ffd179, Oxford Nanopore Technologies) was employed to translate MinION raw reads (FAST5) into quality tagged sequence reads with 4000 reads per FASTQ-file. The barcode trimming option (model version: dna_r9.4.1_450bps_sup.cfg, and dna_r10.4.1_e8.2_400bps_sup) was utilised during the process. The flye software (version 2.8.3) was used to assemble each strain’s quality tagged sequence reads into a complete, circular contig. The assemblies were polished in two stages. First, four iterative rounds of racon (v1.4.21) were conducted with parameters including match 8, mismatch 6, gap 8, and window-lengths of 500. Subsequently, medaka (version 1.4.3) was employed on the last racon-polished assembly using the models r941_min_sup_g507 and r10.4.1_e82_400bps_sup_g615. Finally, Abricate (v1.0.0) was utilised to screen the resulting, corrected assembly for resistance and virulence genes.

The sequence data were submitted to the NCBI database under the BioProject number PRJNA779589.

### 4.5. Genomic DNA Dilution

To validate and quantify the experiments, defined genomic DNA 10-fold dilution series of each reference strain were prepared. The genomic equivalents (GE) were then calculated based on the genome size of the sequenced specimens of the bacterial pathogen species. This allowed for a relative quantification of all marker genes located on the chromosome, including all species markers. The genes responsible for resistance in the various reference strains were located either on the chromosome or plasmid, depending on the strain. For plasmid-encoded resistance marker genes, the calculation provided a semi-quantitative estimation of the copy number based on the genome copy number.

### 4.6. DNA Pre-Amplification

Pre-amplification assays for validation with genomic DNA were carried out in 20 µL reaction volumes containing a final concentration of 200 nM of primer mix, 400 nM primer B, and 2 µL of genomic DNA in 1× PCR buffer B1V7 (BLINK AG, Jena, Germany) and 6 U HotStart-Taq polymerase (Biotechrabbit, Berlin, Germany). The primer mix contained the chimeric oligonucleotides for all targets which were equimolar, mixed with a final concentration of 2 mM. Validation of the method was carried out with dilutions of genomic DNA with 10 and 100 GE/µL as templates. The thermal cycling program consisted of an initial denaturation at 95 °C for 2 min followed by 30 cycles of denaturation for 15 s at 95 °C and annealing and elongation for 45 s at 43 °C.

To investigate the influence of human DNA to performance of the pre-amplification reaction, the method was performed as described above. The pre-amplification reaction mixes contained 200 or 20 GE as the template for each tested strain with an addition of 1 µg of human DNA (Roche, Grenzach-Wyhlen, Germany) with a final concentration of 50 ng (ca. 7700 human GE) per µL as a background to the mix.

The pre-amplification assays of spiked and prepared blood samples were performed in a final volume of 100 µL, with a final concentration of 1 mM for primer B and a template volume of 50 µL. All other parameters and the cycling program were unchanged.

### 4.7. qPCR Assays

For quantification of the pre-amplification, qPCR assays were performed in 20 µL volumes. The qPCR mix contained 1× PCR buffer B1V10 (BLINK AG, Jena, Germany), 3 mM MgCl_2_, 200 nM of each primer and TaqMan probe, 4 U HotStart-Taq polymerase (Biotechrabbit, Berlin, Germany), 1 mg/mL BSA (NEB, Ipswich, MA, USA), 200 nM dNTPs/dUTP (Biotechrabbit, Berlin, Germany), and 0.2 U Uracil-DNA glycosylase (Biotechrabbit, Berlin, Germany). Amplification was carried out in a QuantStudio5 qPCR cycler (Thermo Fisher Scientific Inc., Waltham, MA, USA) with an initial denaturation at 9 °C for 5 min followed by 40 cycles of 20 s denaturation at 95 °C and annealing and elongation at 61 °C for 30 s.

For validation of the monoplex qPCR-assays, a calibration curve was prepared for each marker with a 10-fold dilution series from 10^6^ down to 10^1^ GE/µL with a 2 µL template volume of genomic DNA from reference strains. To check the specificity of all primer/probe sets, cross hybridization experiments were performed with all primer/probe sets and all genomic DNA samples from the non-target reference strains. As a template, 2 µL genomic DNA was used in dilution D4 (1000 cp/µL). The efficiency of the quantitative polymerase chain reaction was determined based on the slope of the calibration curve.

To quantify the copy number of species-specific and resistance markers in the pre-amplified samples, an instant calibration curve was generated for each monoplex qPCR assay. Therefore, the assay included three standard dilutions of genomic DNA (D2 with 10^6^ cps/µL, D4 with 10^4^ cps/µL, and D6 with 10^2^ cps/µL) from the respective reference strain and marker gene, and an assay-specific calibration curve was constructed from the measured CT values of the standard dilutions with known copy numbers.

### 4.8. Multitarget Pre-Amplification

**Figure 6:**
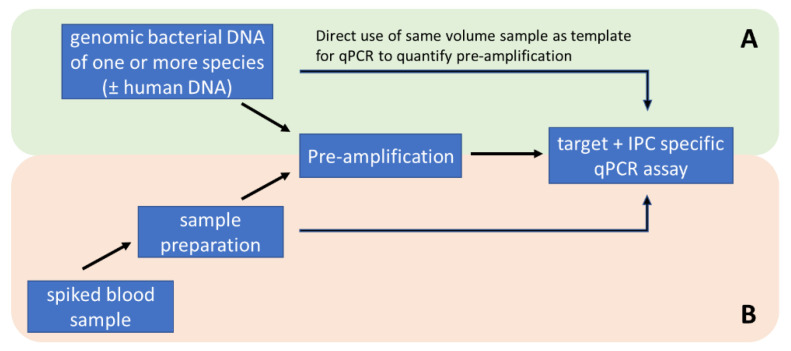
Methodological workflow for the development and optimization of pre-amplification with standardised genomic DNA samples (**A**) and testing of the methodology with artificially spiked whole blood samples (**B**).

To test the feasibility of parallel amplification of potential DNA targets, genomic DNA of all 10 different bacterial species were mixed together at a final concentration of 100 GE/µL. Pre-amplification experiments were performed with 200 GE per reaction. The quantification of the multi-target pre-amplification was performed by single target duplex qPCR assays target and IPC), as described before (Figure 6A).

### 4.9. Blood Sample Preparation

To incorporate an internal process control, a syringe (5 mL BD Syringe REF 309649) was filled with dyed *Bacillus atrophaeus* spores (ATCC 9372). A spore aliquot of 18.8 µL was coloured with 0.05 mM Brilliant Blue (Erioglaucine disodium salt, 861,146 Sigma Aldrich) and dried with 20% Cavasol (2-Hydroxypropyl-beta-cyclodextrin, 778,966 Sigma Aldrich) at room temperature overnight at the bottom of the syringe.

Blood samples (provided by IKTJ gGmbH, Jena, Germany, in accordance with the Transfusion Act) were spiked with the respective type and quantity of bacteria. The syringe with dried IPC was connected to a capped Leucosorb filter (Cap Luer Lock; PALL Acrodisc PSF REF: AP-4952, 8 µm pore size) and 3 mL of spiked blood was pipetted reversely into the syringe. After 5 min of incubation at room temperature, the IPC pellet was dissolved. Then, the blood sample was directly filtered into a falcon tube (15 mL falcon REF 188271-N), containing the BLINK erythrocyte lysis buffer (BEL buffer) (BLINK AG, Jena, Germany), and rotary incubated for 10 min at 30 rpm (Loopster digital, IKA, Staufen, Germany). To separate the bacteria from erythrocyte debris, the entire sample was again filtered through a bacterial filter (VWR syringe filter, 0.45 µm pore size, PES membrane, 25 mm size, 514–1261) that was connected to a 10 mL syringe (BD Syringe REF 300912). The filtrate was pushed directly into a new falcon tube. The following washing step with 3 mL PBS, using a fresh syringe (5 mL BD Syringe REF 309649) was also pushed into the falcon tube, which was dumped afterwards. Remaining buffer was removed via air pressure. In the last step, the bacterial filter was flushed backwards. For this, the bacterial filter was rotated 180° and directly connected to a fresh syringe (3 mL BD Syringe REF 309658) containing 1 mL PBS solution via an adapter (PP-LF-LF). The backwash sample with the enriched bacteria was transferred into the lysis tube, containing 400 mg ceramic beads (zirconium silicate beads, Biolabproducts GmbH, Bebensee, Germany). The lysis tube can be stored for up to one week.

For DNA purification and to concentrate the DNA, the lysis tube with the enriched bacterial suspension was placed into the tube holder of the BLINK X Shaker and ran thrice at 3000 rpm for 60 s. Following DNA purification, steps were performed with the MagaZorb^®^ DNA Mini-Prep Kit. A total of 750 µL of the lysed bacteria sample was pipetted on top of 75 µL proteinase K solution and mixed by pipetting up and down. After adding 750 µL lysis buffer, the sample was mixed well and incubated for 10 min, 500 rpm, at 56 °C. The bacterial DNA was subsequently bound to magnetic beads by adding 1875 µL binding buffer and 40 µL beads, and then incubated for 30 min at a rotary mixer. The magnetic beads were washed twice with 1 mL wash buffer. Finally, the DNA was eluted in 75 µL pure water and then incubated for 20 min at 600 rpm and 30 °C.

Isolated, purified, and concentrated bacterial DNA was directly analysed via quantitative PCR or pre-amplified. Quantitative PCR ran with 2 µL DNA eluate in 18 µL of PCR-Mastermix twice (technical replicate) and pre-amplification (PA) was performed with 50 µL DNA eluate in 50 µL PA-Mastermix. To optimise the storage conditions, the 100 µL PA was filled with 50 µL of 0.3% PEG6000 solution. Subsequently, PA was analysed via quantitative PCR and run with 2 µL PA as a template in 18 µL of PCR-Mastermix in two technical replicates (Figure 6B).

### 4.10. Data Analysis Spiked Blood Samples

The datasets comprising Ct values for various samples were analyzed to assess the differences between two experimental methods, denoted as ‘qP’ (qPCR) and ‘PA’ (qPCR with pre-amplification). The raw data, initially containing Ct values and ‘not detected’ (n.d.) entries, were pre-processed. The ‘n.d.’ entries, representing undetected or below detection limit values, were converted to NaN (not a number) to facilitate numerical calculations. For each sample, descriptive statistics were computed. This included the calculation of mean, median, and standard deviation (Std Dev) for both the qP and PA methods. These measures provide insights into the central tendency and variability of the Ct values across different experimental conditions. To determine the statistical significance of the differences in Ct values obtained by the qP and PA methods, paired t-tests were performed. Each sample measured by both methods was treated as a pair, and only pairs with complete data (i.e., non-missing values in both methods) were included in the analysis. The t-test results, comprising the t-statistic and the ^p^-value, were reported for each sample. A low *p*-value (typically < 0.05) indicates a statistically significant difference between the two methods for that particular sample. In cases where a sample lacked sufficient data (e.g., excessive missing values in either method), those samples were excluded from the t-test analysis to maintain statistical robustness. The analyses were conducted using Python, with the Pandas library for data manipulation and the SciPy library for statistical tests. The resulted statistical data according to Figure 5 were combined in the supplementary Table A5 for each sample.

## 5. Conclusions

Direct molecular analysis of bloodstream infections from full blood samples presents challenges due to the high human DNA background, PCR inhibitors, low pathogen DNA concentration, and large sample volumes. The study aimed to overcome these limitations by introducing a sample preparation method that significantly improved target-specific DNA amplification. Filtration steps effectively minimised human material, enhancing performance without negatively impacting amplification. The pre-amplification method, which includes an artificial sequence, is robust, stable, and highly sensitive. This enables the detection of pathogens at a critical threshold of 1 CFU/mL in blood, which is a significant advancement for sepsis diagnosis. The effectiveness of the sample preparation pipeline for real clinical blood samples is yet to be determined, but it will be tested in an upcoming clinical study. The pre-amplification method is versatile and allows for the amplification of a panel of molecular species and resistance markers simultaneously. Although false-positives are possible, the study recommends using a closed system and sterile components to mitigate contamination risks, which is a crucial step towards early sepsis diagnosis and bypasses time-consuming cultivation methods.

## Figures and Tables

**Table 1:** Comparison of pre-amplification results between bacterial DNA as the sole target and bacterial DNA spiked with 1 µg of human DNA per reaction as an artificial sample matrix. Notably, the presence of human DNA in the sample matrix results in a significant reduction in bacterial DNA genomic equivalent (GE) copy numbers, with decreases of two to three orders of magnitude.

Initial Concentration of Target-DNA			2 × 10^2^ GE		2 × 10^1^ GE	
Background DNA			IPC	IPC/human	IPC	IPC/human
			Calculated Concentration after Pre-Amplification			
Species		Marker	GE/rxn	GE/rxn	GE/rxn	GE/rxn
*A. baumannii*	Species	*basC*	3.5 × 10^8^	2.5 × 10^5^	6.1 × 10^7^	2.0 × 10^4^
*E. coli*		*gad*	1.8 × 10^8^	1.5 × 10^5^	2.8 × 10^7^	4.4 × 10^3^
*P. aeruginosa*		*ecfX*	2.2 × 10^7^	4.8 × 10^3^	4.5 × 10^6^	4.2 × 10^3^
*C. freundii*		*cfa*	2.8 × 10^7^	1.7 × 10^5^	1.3 × 10^6^	9.0 × 10^3^
*K. pneumoniae*		*khe*	3.3 × 10^7^	2.4 × 10^5^	4.8 × 10^5^	7.4 × 10^4^
*S. aureus*		*gapA*	4.5 × 10^7^	1.6 × 10^4^	1.3 × 10^6^	8.35 × 10^3^
*E. faecium*		*aac6li*	7.5 × 10^7^	4.9 × 10^5^	5.8 × 10^6^	5.5 × 10^4^
*E. faecalis*		*ddl*	4.4 × 10^9^	2.6 × 10^6^	3.3 × 10^8^	2.2 × 10^5^
*S. pneumoniae*		*lytA2*	6.3 × 10^8^	6.7 × 10^5^	6.4 × 10^7^	2.4 × 10^4^
*S. epidermidis*		*sesC*	9.3 × 10^7^	8.4 × 10^5^	8.8 × 10^6^	4.7 × 10^5^
*K. pneumoniae*	Resistance	*bla*OXA-48	4.5 × 10^8^	5.7 × 10^5^	7.7 × 10^7^	7.8 × 10^4^
*E. coli*		*bla*CTX-M-9	8.7 × 10^7^	9.8 × 10^4^	1.1 × 10^7^	6.1 × 10^3^
*E. coli*		*bla*KPC	3.6 × 10^9^	8.1 × 10^5^	3.9 × 10^8^	2.0 × 10^4^
*C. freundii*		*bla*VIM	5.3 × 10^7^	8.0 × 10^5^	1.5 × 10^7^	1.6 × 10^5^
*K. pneumoniae*		*bla*NDM	3.3 × 10^8^	6.3 × 10^5^	7.6 × 10^7^	7.9 × 10^4^
*K. pneumoniae*		*bla*CTX-M-15	4.4 × 10^8^	3.4 × 10^6^	5.6 × 10^7^	3.5 × 10^5^
*S. aureus*		*mecA*	1.4 × 10^7^	9.6 × 10^4^	5.7 × 10^5^	4.7 × 10^3^
*E. faecium*		*vanA*	2.9 × 10^8^	2.6 × 10^6^	4.0 × 10^7^	4.7 × 10^5^
*E. faecium*		*vanB*	4.9 × 10^7^	4.6 × 10^5^	2.2 × 10^6^	2.5 × 10^4^
*A. baumannii*		*bla*OXA-23	9.9 × 10^8^	1.6 × 10^6^	1.5 × 10^8^	3.6 × 10^5^
*A. baumannii*		*bla*OXA-58	1.3 × 10^9^	2.4 × 10^6^	9.8 × 10^7^	5.8 × 10^5^

**Table 2:** Panel of target genes used for the present study.

Description	Species/Target	Gene	Function	Reference
Species	*Klebsiella pneumoniae*	*khe*	Hemolysin	AF293352.1 [91:579]
Species	*Acinetobacter baumannii*	*basC*	Acinetobactin biosynthesis	AY571146.1 [6565:7875]
Species	*Escherichia coli*	*gad*	Glutamate decarboxylase	AE014075.1 [1756318:1757787]
Species	*Pseudomonas aeruginosa*	*ecfX*	Extracellular sigma factor	DQ996558.1 [1:528]
Species	*Citrobacter freundii*	*cfa*	Colicin biosynthesis	U09771.1 [1:271]
Species	*Staphylococcus epidermidis*	*atlE*	Autolysin	U71377.1 [2620:6627]
Species	*Staphylococcus epidermidis*	*sesC*	Surface protein	AE015929.1 [2291621:2293651]
Species	*Staphylococcus aureus*	*gapA*	Glyceraldehyde-3-phosphate dehydrogenase	CP007176.1 [863178:864188]
Species	*Enterococcus faecium*	*aac6Ii*	6′-*N*-aminoglycoside acetyltransferase	L12710.1 [1:1485]
Species	*Enterococcus faecalis*	*ddl*	*D*-alanine ligase	AB186053.1 [1:1071]
Species	*Streptococcus pneumoniae*	*lytA*	Autolysin	HG514154.1 [1:957]
Resistance	Modified staphylococcal penicillin-binding protein, PBP2a	*mecA*	Methicillin resistance	AY786579 [1:2007]
Resistance	Carbapenemase	*bla*KPC	Class B metallo-beta-lactamase	EU447304.1 [15:896]
Resistance	Carbapenemase	*bla*NDM	Class B metallo-beta-lactamase	FN396876.1 [2407:3219]
Resistance	Carbapenemase	*bla*VIM	Class B metallo-beta-lactamase	Consensus
Resistance	Carbapenemase	*bla*OXA-48	Class D beta-lactamase	Consensus (OXA-48-group)
Resistance	Carbapenemase	*bla*OXA-181	Class D beta-lactamase	
Resistance	Carbapenemase	*bla*OXA-23	Class D beta-lactamase	AJ132105.1 [972:1793]
Resistance	Carbapenemase	*bla*OXA-58	Class D beta-lactamase	AY665723.1 [3301:4143]
Resistance	ESBL	*bla*CTX-M15	Extended-spectrum beta-lactamase	AB698966 [1:895]
Resistance	ESBL	*bla*CTX-M9	Extended-spectrum beta-lactamase	AF174129.3 [6336:7211]
Resistance	D-alanine-D-alanine ligase	*vanA*	Vancomycin resistance	AF516335.1 [3748:4779]
Resistance	D-alanine-D-alanine ligase	*vanB*	Vancomycin resistance	Z83305.1 [150:1178]

**Table 3:** List of chimeric oligonucleotides for pre-amplification.

Nr.	Name	Sequence (5′-3′)
1	aac6_rnd_fw	GTTTCCCAGTCACGATCTGGCCGGAAGA
2	aac6_rnd_rev	GTTTCCCAGTCACGATCCCTCTGGAAGCTAC
3	atlE_rnd_fw	GTTTCCCAGTCACGATCGCTGCCACACT
4	atlE_rnd_rev	GTTTCCCAGTCACGATCACCAGATTTACCG
5	basC_rnd_fw	GTTTCCCAGTCACGATCCGATGGCATGAA
6	basC_rnd_rev	GTTTCCCAGTCACGATCCTCCAAATCGACA
7	cfa_rnd_fw	GTTTCCCAGTCACGATCGGGAAAAGAGCTG
8	cfa_rnd_rev	GTTTCCCAGTCACGATCCGCAAAGAGATCG
9	ctx15_rnd_fw	GTTTCCCAGTCACGATCAAATCACTGCGC
10	ctx15_rnd_rev	GTTTCCCAGTCACGATCATCAATGCCACAC
11	ctx9_rnd_fw	GTTTCCCAGTCACGATCCGCGTTGCAGTA
12	ctx9_rnd_rev	GTTTCCCAGTCACGATCCCCAGCGTAAGC
13	ddl_rnd_fw	GTTTCCCAGTCACGATCGATGCCCGAGCA
14	ddl_rnd_rev	GTTTCCCAGTCACGATCAGCCACTTCCATC
15	ecfX_rnd_fw	GTTTCCCAGTCACGATCATGGATGAGCG
16	ecfX_rnd_rev	GTTTCCCAGTCACGATCCTGCCCAGGTG
17	gad_rnd_fw	GTTTCCCAGTCACGATCTGGCTCCGCTG
18	gad_rnd_rev	GTTTCCCAGTCACGATCAGGCGCAGGAATT
19	gapA_rnd_fw	GTTTCCCAGTCACGATCGAAGCAGGCGC
20	gapA_rnd_rev	GTTTCCCAGTCACGATCGTGAGGTGCGTC
21	IPC_BG_rnd_fwd	GTTTCCCAGTCACGATCCGTGTATTTAACGT
22	IPC_BG_rnd_rev	GTTTCCCAGTCACGATCTTTTACCTACACCG
23	khe_rnd_fwd_2	GTTTCCCAGTCACGATCAAGGGCCCGA
24	khe_rnd_rev_2	GTTTCCCAGTCACGATCTCCTGGCGCGT
25	kpc_rnd_fw	GTTTCCCAGTCACGATCGCCGTCTAGTTC
26	kpc_rnd_rev	GTTTCCCAGTCACGATCATGGAGCCGCC
27	lytA_rnd_fw	GTTTCCCAGTCACGATCCAGACCGCTGGAA
28	lytA_rnd_rev	GTTTCCCAGTCACGATCGGTAGTACCAGCC
29	mecA_rnd_fw	GTTTCCCAGTCACGATCGCACACCTTCATA
30	mecA_rnd_rev	GTTTCCCAGTCACGATCGCCAACCTTTACC
31	ndm_rnd_fw	GTTTCCCAGTCACGATCCTCGACATGCCG
32	ndm_rnd_rev	GTTTCCCAGTCACGATCGATGCGCGTGAG
33	oxa23_rnd_fw	GTTTCCCAGTCACGATCTCAGGTGATTCATC
34	oxa23_rnd_rev	GTTTCCCAGTCACGATCGCGGTAAATGACC
35	oxa48_rnd_fw	GTTTCCCAGTCACGATCCCGCATCTACCT
36	oxa48_rnd_rev	GTTTCCCAGTCACGATCTGGCGATATCGC
37	oxa58_rnd_fw	GTTTCCCAGTCACGATCTGCCAATGCACTA
38	oxa58_rnd_rev	GTTTCCCAGTCACGATCCAACTTCCGTGC
39	sesC_rnd_fw	GTTTCCCAGTCACGATCTGAAGAGAACAGATA
40	sesC_rnd_rev	GTTTCCCAGTCACGATCGATATCTGCGTCAG
41	vanA_rnd_fw	GTTTCCCAGTCACGATCGTACTCTCGCCG
42	vanA_rnd_rev	GTTTCCCAGTCACGATCCGCAACGATGTAT
43	vanB_rnd_fw	GTTTCCCAGTCACGATCCGGTGTATGGAAG
44	vanB_rnd_rev	GTTTCCCAGTCACGATCCCTGTATCGCACC
45	vim_rnd_fw	GTTTCCCAGTCACGATCGACGACCGCGT
46	vim_rnd_rev	GTTTCCCAGTCACGATCGTCGGTCGAATGC
47	Primer B	GTTTCCCAGTCACGATC

**Table 4:** List of primers and probes for monoplex qPCR assays.

Nr.	Name	Sequence 5′-3′	Length	GC Content in %
1	aac6Ii_fw_2	TCGGCAGAAGAAGTAGAAGA	20	45.0
2	aac6Ii_probe_2	ATTGGTGCAATCCCTCAATACGGTATCACA	30	43.3
3	aac6Ii_rev_2	ACTAATGGATGCAATTCCCAA	21	38.1
4	atlE_fwd_3	CTGGTACAAATTATGGTTGGGT	22	40.9
5	atlE_probe_3	GTACCTTGGGGCACATATAATCAAGTGGC	29	48.3
6	atlE_rev_3	CACTGTACCATAAAGATATGTTGC	24	37.5
7	basC_fw	CTTGGTTACTATGGCCAATCC	21	47.6
8	basC_probe	CCACGCCGTGAATATGACCATTATTG	26	46.2
9	basC_rv	GGTAATTGTTTTGAAGCCCA	20	40.0
10	cfa_fw	CTGGGACATTCAACTTCATC	20	45.0
11	cfa_probe	TAGGGCTTGGCGAAAGCTATATGGAA	26	46.2
12	cfa_rv	TCAGGATTTTGCAGAACAGAA	21	38.1
13	ctx-M15_fw	CAGTTCACGCTGATGGC	17	58.8
14	ctx-M15_probe	ACCGTCACGCTGTTGTTAGGAAGTGT	26	50.0
15	ctx-M15_rv	CGACTGCCGCTCTAATTC	18	55.6
16	ctx-M9_fw_2	CGCCATGAACAAATTGATTGC	21	42.9
17	ctx-M9_probe_1	TCGGCGATGAGACGTTTCGTCTGG	24	58.3
18	ctx-M9_rv	GGAATGGCGGTATTCAGC	18	55.6
19	ddl_fw2	TTAGGAAATGAAGATGTCCGTAC	23	39.1
20	ddl_probe2	TTACCTGGTGAAGTGGTGAAAGATGTCG	28	46.4
21	ddl_rv2	GCTACTTCTTCTGGAACATGC	21	47.6
22	ecfX_fw	ATGAGCGCTTCCGTGGTTC	19	57.9
23	ecfX_probe	TCTCGCATGCCTATCAGGCGTTCCAT	26	53.9
24	ecfX_rv	AGGAAGCGCAGCAACTCG	18	61.1
25	gad_fw2	CTGGGTTATCTGGCGTGA	18	55.6
26	gad_probe_2	AAGAAGCGCTGCCGCAGGAACTG	23	60.9
27	gad_rv2	GCGGGAGAAGTTGATGG	17	58.8
28	gapA_fw2	GGTGACTTAAAAACAATCGTATTCA	25	32.0
29	gapA_probe2	GGTTCTGAAACAGTTGTTTCAGGTGCTTCA	30	43.3
30	gapA_rv2	CTTCAACTAAACCAAAGTCATCG	23	39.1
31	IPC_BG_fwd	GCGGCAAACACGGAGAAA	18	55.6
32	IPC_BG_probe	CCGATTCACAGACAAGCTCCGTCATTTGATC	31	48.4
33	IPC_BG_rev	TCCACCGAACAATCCGATC	19	52.6
34	khe_fw_2	GGTTTACGTCTCAACCGG	18	55.6
35	khe_probe_2	TGAGGAAGAGTTCATCTACGTGCTGGAGGG	30	53.3
36	khe_rv	AGAGATAGCCGTTTATCCACAC	22	45.5
37	kpc_fw	CTTGTCTCTCATGGCCG	17	58.8
38	kpc_probe	TGCCACCGCGCTGACCAACCT	21	66.7
39	kpc_rv	AGTTTAGCGAATGGTTCCG	19	47.7
40	lytA_fw2	GCTGGAAGAAAATCGCTG	18	50.0
41	lytA_probe2	GACAGGCTGGGTCAAGTACAAGGACAC	27	55.6
42	lytA_rv2	TTCCGTCCGCTGACTG	16	62.5
43	mecA_fw2	TGGCATGAGTAACGAAGAATATAA	24	33.3
44	mecA_probe2	AAAGAACCTCTGCTCAACAAGTTCCAGA	28	42.9
45	mecA_rv2	GAGTTGAACCTGGTGAAGTTG	21	47.6
46	ndm_fw2	GGTTTGATCGTCAGGGATG	19	52.6
47	ndm_probe2	ATGACCAGACCGCCCAGATCCTCA	24	58.3
48	ndm_rv2	GACCGGCAGGTTGATCT	17	58.8
49	oxa-23_fw	TCAGGTGTGCTGGTTATTCAAA	22	40.9
50	oxa-23_probe_611	CTAAGCCGCGCAAATACAGAATATGTGCC	29	48.3
51	oxa-23_rv	CGATCAGGGCATTCAACATT	20	45.0
52	oxa-48_fw	TTCCCAATAGCTTGATCGC	19	47.4
53	oxa-48_probe	TCGATTTGGGCGTGGTTAAGGATGAAC	27	48.2
54	oxa-48_rv	CCATCCCACTTAAAGACTTGG	21	47.6
55	oxa58_fw	TTAAGTGGGATGGAAAGCC	19	47.4
56	oxa58_probe	GCCATGCAAGCATCTACAGTGCCTG	25	56.0
57	oxa58_rv	GCAATTCACTTTGCATTAAGCT	22	36.4
58	sesC_fw	GTGTCTACCTCAAGCTGTCATG	22	50.0
59	sesC_probe	TTAGTGGTTCGCTGGTTGGTTATGGCTT	28	46.4
60	sesC_rv	TTGGATTTTGTCAGCGATG	19	42.1
61	vanA_fwd	TCAGCTTTGCATGGCAAG	18	50.0
62	vanA_probe	CCATACAAGGTCTGTTTGAATTGTCCGG	28	46.4
63	vanA_rv	GCTGAGCTTTGAATATCGCA	20	45.0
64	vanB_fwd_2	GCCATGTACGGAATGGGAAG	20	55.0
65	vanB_probe_2	CCCGCCATACTCTCCCCGGATAGGAA	26	61.5
66	vanB_rev_2	CAAAACCGGGAAAGCCAC	18	55.6
67	vim_fw	GGCAACGTACGCATCAC	17	58.8
68	vim_probe	TCTCTAGAAGGACTCTCATCGAGCGGG	27	55.6
69	vim_rv	GCAGCACCGGGATAGAA	17	58.8

**Table 5:** Bacterial strains containing resistance genes.

Organism	Strain ID	Resistance Genes (as Identified by the WGS Tool Abricate)
*Acinetobacter baumannii*	215784	*bla*VIM-2
*Acinetobacter baumannii*	240611	*bla*NDM-1; *bla*NDM2
*Acinetobacter baumannii*	95932	*bla*OXA-58
*Acinetobacter baumannii*	301751	*bla*NDM, *bla*OXA-23-like
*Acinetobacter baumannii*	303315	*bla*OXA-58
*Bacillus atrophaeus*	97424	-
*Citrobacter freundii*	240619	*bla*VIM, *bla*OXA-48
*Citrobacter freundii*	279615	*bla*VIM
*Enterococcus faecalis*	95737	-
*Enterococcus faecium*	95735	vanA
*Enterococcus faecium*	95738	vanB
*Escherichia coli*	240608	*bla*OXA-48, *bla*CTX-M-9
*Escherichia coli*	240615	*bla*KPC-2
*Escherichia coli*	240780	*bla*VIM-4, *bla*CTX-M-1/15
*Escherichia coli*	296351	*bla*NDM-1, *bla*NDM2, *bla*CTX-M-1/15
*Escherichia coli*	319495	*bla*CTX-M-9
*Klebsiella pneumoniae*	239644	*bla*OXA-48-like, *bla*CTX-M-1/15
*Klebsiella pneumoniae*	240799	*bla*NDM-1, *bla*OXA-181/232, *bla*CTX-M-1/15
*Klebsiella pneumoniae*	280236	*bla*CTX-M-1/15
*Klebsiella pneumoniae*	272567	*bla*VIM-1
*Klebsiella pneumoniae*	274401	*bla*KPC-2
*Pseudomonas aeruginosa*	279584	*bla*VIM
*Staphylococcus aureus*	95430	mecA
*Staphylococcus epidermidis*	95428	mecA
*Streptococcus pneumoniae*	95736	-

## Data Availability

All relevant data are provided as supplementary files. The sequences of the reference strain genomes can be accessed under the BioProject accession number PRJNA779589.

## Appendix A

**Table A1:** Validation of qPCR monoplex assays was performed using 10-fold standard dilutions of genomic DNA as a template, ranging from 10^6^ GE/µL down to 10^1^ GE/µL. Efficiency was calculated based on the slope of the calibration curve.

	Target	Efficiency (%)	R^2^
Species	*basC*	94.57	>0.9996
	*gad*	84.03	>0.9937
	*khe*	102.41	>0.9984
	*cfa*	81.26	>0.9758
	*ecfX*	93	>0.9964
	*gapA*	93.87	>0.9995
	*aac6li*	108.72	>0.9828
	*ddl*	95.21	>0.9975
	*lytA*	97.28	>0.9994
Resistance	*bla*OXA-48	98.06	>0.9929
	*bla*NDM	88.69	>0.9953
	*bla*VIM	92.64	>0.9884
	*bla*CTX-M9	90.07	>0.9934
	*bla*CTX-M15	88.35	>0.9953
	*bla*KPC	93.69	>0.9998
	*mecA*	87.8	>0.9995
	*vanA*	100.15	>0.9988
	*vanB*	88.87	>0.9988
	*bla*OXA-58	102.44	>0.9984

**Table A2:** Results of the cross-hybridization experiments with the species marker.

Strain	Target	*basC*	*cfa*	*ecfX*	*gad*	*gapA*	*khe*	*aac6*	*msrC*	*ddl*	*lytA*	*lytA2*	*atlE*	*sesC*
*Acinetobacter baumannii*	*basC*	P	n	n	n	n	n	n	n	n	n	N	n	n
*Citrobacter freundii*	*cfa*	n	P	n	n	n	n	n	n	n	n	N	n	n
*Pseudomonas aeruginosa*	*ecfX*	n	n	P	n	n	n	n	n	n	n	N	n	n
*Escherichia coli*	*gad*	n	n	n	P	n	n	n	n	n	n	N	n	n
*Staphylococcus aureus*	*gapA*	n	n	n	n	P	n	n	n	n	n	N	n	n
*Klebsiella pneumoniae*	*khe*	n	n	n	n	n	P	n	n	n	n	N	n	n
*Enterococcus faecium*	*aac6li*, *msrC*	n	n	n	n	n	n	P	P	n	n	N	n	n
*Enterococcus faecalis*	*ddl*	n	n	n	n	n	n	n	n	P	n	N	n	n
*Streptococcus pneumoniae*	*lytA*, *lytA2*	n	n	n	n	n	n	n	n	n	P	P	n	n
*Staphylococcus epidermidis*	*atlE*, *sesC*	n	n	n	n	n	n	n	n	n	n	N	P	P
		n	no qPCR signal (expected)			P	positive control				y	signal (not expected)		

**Table A3:** Results of the cross-hybridization experiments with resistance marker.

Strain	Target	*bla*VIM	*bla*OXA-48	*bla*OXA-181	*bla*NDM-2	*bla*CTX-M-15	*bla*CTX-M-9	*aac6*	*mecA*	*vanA*	*vanB*	*bla*OXA-58	*bla*OXA*-*23
*Acinetobacter baumannii*	*bla*VIM-2	P	n	n	n	n	n	n	n	n	N	n	n
*Klebsiella pneumoniae*	*bla*OXA-48-like, *bla* CTX-M-1/15	n	P	n	n	P	n	n	n	n	N	n	n
*Escherichia coli*	*bla*OXA-48-like, *bla*CTX-M-9	n	0	n	n	n	P	n	n	n	N	n	n
*Acinetobacter baumannii*	*bla*NDM-1	n	n	n	P	n	n	n	n	n	N	n	n
*Escherichia coli*	*bla*KPC-2, *bla*CTX-M-9	n	n	n	n	n	0	P	n	n	N	n	n
*Citrobacter freundii*	*bla*VIM, *bla*OXA-48-like	0	0	n	n	n	n	n	n	n	N	n	n
*Escherichia coli*	*bla*VIM-4, *bla*CTX-M-1/15	0	n	n	n	0	n	n	n	n	N	n	n
*Klebsiella pneumoniae*	*bla*NDM-1, *bla*OXA-181/232, *bla*CTX-M-1/15	n	0	P	0	0	n	n	n	n	N	n	n
*Pseudomonas aeruginosa*	*bla*VIM	0	n	n	n	n	n	n	n	n	N	n	n
*Citrobacter freundii*	*bla*VIM	0	n	n	n	n	n	n	n	n	N	n	n
*Klebsiella pneumoniae*	*bla*CTX-M-1/15	n	n	n	n	0	n	n	n	n	N	n	n
*Escherichia coli*	*bla*NDM-1, *bla*VIM, *bla*CTX-M-1/15	P	n	n	0	0	n	n	n	n	N	n	n
*Staphylococcus aureus*	mecA	n	n	n	n	n	n	n	P	n	N	n	n
*Klebsiella pneumoniae*	*bla*VIM-1	0	n	n	n	n	n	n	n	n	N	n	n
*Klebsiella pneumoniae*	*bla*KPC-2, *bla*CTX-M-2	n	n	n	n	n	n	0	n	n	N	n	n
*Enterococcus faecium*	vanA	n	n	n	n	n	n	n	n	P	N	n	n
*Enterococcus faecalis*	−	n	n	n	n	n	n	n	n	n	N	n	n
*Enterococcus faecium*	vanB	n	n	n	n	n	n	n	n	n	P	n	n
*Escherichia coli*	*bla*CTX-M-9	n	n	n	n	n	0	n	n	n	N	n	n
*Streptococcus pneumoniae*	−	n	n	n	n	n	n	n	n	n	N	n	n
*Acinetobacter baumannii*	*bla*OXA-58	n	n	n	n	n	n	n	n	n	N	P	n
*Acinetobacter baumannii*	*bla*NDM2, *bla*OXA-23-like	n	n	n	0	n	n	n	n	n	N	n	P
*Acinetobacter baumannii*	*bla*OXA-58	n	n	n	n	n	n	n	n	n	N	0	n
*Staphylococcus epidermidis*	−	n	n	n	n	n	n	n	P	n	N	n	n
		n	no qPCR signal (expected)				P	positive control					
		0	not tested				y	qPCR signal (not expected)					

**Table A4:** Commercial molecular sepsis diagnostics currently available on the market and their parameters.

Manufacturer	Assay	Method	Sample	Sample Volume (mL)	Sample Preparation	Sample-to-Result-Time (h)	LOD (CFU/mL)	Sensitivity/ Specificity (%)	Range of Detection	Detection of Polymicrobial Infections	AMR Marker	References
Abbott Molecular	IRIDICA BAC BSI	PCR and ESI-MS	Whole blood	5.0	External (IRIDICA BB/SP)	6	0.25–128	45–83/69–94	780 bacteria and *Candida* spp.	Yes	*mecA*, *vanA*, *vanB*, and *bla*KPC	[31,32,33]
Roche Molecular Diagnostics	LightCycler^®^ SeptiFast Test	Multiplex real-time PCR with DNA-DNA hybridization and melting curves	Whole blood	1.5	External (MagNA Lyser)	4–6	3–100	63–83/83–95	16 bacteria, *Candida*, and *Aspergillus fumigatus*	Yes	*mecA*	[31,32]
Molzym	SepsiTest	Universal PCR and sequencing	Whole blood	1.0	External	8–10	10–80	11–87/83–96	345 bacteria and 13 fungi	n.a	None	[31,32,34]
Immunexpress	SeptiCyte	“Host-response gene-expression assay”—quantitative RT-qPCR of four RNA biomarker	Whole blood	2.5	External (total RNA)	6	n.a.	n.a./95	Non-direct detection of pathogens	n.a.	None	[23,35,36]
SIRS-Lab	VYOO	Multiplex PCR + Gel-electrophoresis	Whole blood	5.0	External	8	5-100	38–60/72–75	14 Gram-positive, 18 Gram-negative bacteria, 7 fungi	n.a.	*mecA*, *vanA, vanB*, *vanC*, and *bla*SHV	[37,38]
Seegene	Magicplex™ Sepsis Real-time Test	Multiplex real-time PCR	Whole blood	1.0	External (standard kits)	3	n.a.	n.a.	21 bacterial species, 90 bacterial genera, 6 fungi species	Yes	*mecA, vanA/B*	[39,40]
T2 Biosystems	T2Candida and T2Bacteria	Miniaturised magnet-resonance technology	Whole blood	n.a.	Integrated	3–5	10	95/98	3 Gram-negative, 2 Gram-positive bacteria, *Candida* spp.	No	None	[41,42,43,44]
Noscendo	NGS as a service	Sequencing of isolated DNA from whole blood	Whole blood	10.0	External (standard kits)	24	n.a.	n.a.	All pathogens with known sequences (BLAST)	Yes	All resistance genes with known sequences (BLAST)	[45,46,47]
Cepheid	Xpert^®^ MRSA/SA BC	Multiplex real-time PCR	Blood culture	1.0	Integrated	1	150	99/99	MRSA	No	*spa*, *mecA*, *SCCmec*	[48,49]
Curetis	BCU (blood culture application)	Multiplex-PCR with microarray detection via DNA–DNA hybridisation	Blood culture	n.a.	Integrated	4–5	1-10	96/99	10 Gram-positive, 15 Gram-negative bacteria, *M. tuberculosis*, 8 fungi	Yes	16 resistance genes	[50]
bioMérieux Clinical Diagnostics	FilmArray™ Blood culture Panel	Nested PCR: multiplex PCR for enrichment and singleplex PCR for specific detection of targets	Blood culture	n.a.	Integrated	1	n.a.	89–91/100	6 Gram-positive, 10 Gram-negative bacteria, 5 *Candida* spp.	n.a.	*mecA*, *vanA/B*, *bla*KPC	[51,52]
Luminex	GP-BC (Gram-positive) and GN-BC (Gram-negative)	DNA–DNA hybridisation microarray (NanoGrid)	Blood culture	n.a.	Integrated	2	n.a	96–100/98	9 Gram-positive (GP-BC), 5 Gram-negative bacteria (GN-BC)	n.a.	*mecA*, *vanA/B*, CTX-M, IMP, KPC, NDM, OXA, VIM	[53,54]
AdvanDx	PNA FISH/QuickFISH	FISH	Blood culture	0.1	Direct measurement	0.5	10^5^	97–100/89–100	*Staphylococcus aureus* and coagulase-negative Staphylococci	Yes	None	[55,56,57]

**Table A5:** Statistics of the spiked blood sample experiments. Samples with or without pre-amplification were compared pairwise. *p*-values are missing due to incomplete pairs or insufficient parallels for calculating the mean value of a part of the pair. Mean_ORIG_—mean of non-pre-amplified samples, SD_ORIG_—standard deviation of non-pre-amplified samples, Mean_PA_—mean of pre-amplified samples, and SD_PA_—standard deviation of non-pre-amplified samples; blood donors 1 and 2 are BD1 and BD2.

Number	Species	Target	CFU	Blood Donor	Pairs	Mean_ORIG_	SD_ORIG_	Mean_PA_	SD_PA_	t_Statistic	*p*-Value
1	*E. coli*	*gad*	2828	BD1	gad_BD1_2828cfu	33.300	0.368	16.820	0.000	63.385	0.010
2	*E. coli*	*gad*	283	BD1	gad_BD1_283cfu	35.950	0.721	16.165	0.106	45.483	0.014
3	*E. coli*	*gad*	28	BD1	gad_BD1_28cfu	36.530		19.260			
4	*E. coli*	*gad*	3	BD1	gad_BD1_3cfu						
5	*E. coli*	*gad*	0	BD1	gad_BD1_0cfu						
6	*E. coli*	*gad*	2828	BD2	gad_BD2_2828cfu	32.675	0.021	17.680	0.806	27.018	0.024
7	*E. coli*	*gad*	283	BD2	gad_BD2_283cfu	35.305	0.813	15.335	0.021	35.661	0.018
8	*E. coli*	*gad*	28	BD2	gad_BD2_28cfu						
9	*E. coli*	*gad*	3	BD2	gad_BD2_3cfu						
10	*E. coli*	*gad*	0	BD2	gad_BD2_0cfu						
11	*E. coli*	OXA48	2828	BD1	OXA48(gad)_BD1_2828cfu	35.825	0.233	19.365	0.134	235.143	0.003
12	*E. coli*	OXA48	283	BD1	OXA48(gad)_BD1_283cfu	36.830		20.330			
13	*E. coli*	OXA48	28	BD1	OXA48(gad)_BD1_28cfu						
14	*E. coli*	OXA48	3	BD1	OXA48(gad)_BD1_3cfu						
15	*E. coli*	OXA48	0	BD1	OXA48(gad)_BD1_0cfu						
16	*E. coli*	OXA48	2828	BD2	OXA48(gad)_BD2_2828cfu	34.960	0.693	20.055	0.021	31.379	0.020
17	*E. coli*	OXA48	283	BD2	OXA48(gad)_BD2_283cfu	36.135	0.361	20.155	0.247	199.750	0.003
18	*E. coli*	OXA48	28	BD2	OXA48(gad)_BD2_28cfu						
19	*E. coli*	OXA48	3	BD2	OXA48(gad)_BD2_3cfu						
20	*E. coli*	OXA48	0	BD2	OXA48(gad)_BD2_0cfu						
21	*E. coli*	CTX-M9	2828	BD1	CTX-M9(gad)_BD1_2828cfu	34.685	0.587	19.510	0.028	34.885	0.018
22	*E. coli*	CTX-M9	283	BD1	CTX-M9(gad)_BD1_283cfu	36.805	0.940	18.185	0.177	23.570	0.027
23	*E. coli*	CTX-M9	28	BD1	CTX-M9(gad)_BD1_28cfu						
24	*E. coli*	CTX-M9	3	BD1	CTX-M9(gad)_BD1_3cfu						
25	*E. coli*	CTX-M9	0	BD1	CTX-M9(gad)_BD1_0cfu						
26	*E. coli*	CTX-M9	2828	BD2	CTX-M9(gad)_BD2_2828cfu	33.675	0.304	21.220	0.467	108.304	0.006
27	*E. coli*	CTX-M9	283	BD2	CTX-M9(gad)_BD2_283cfu	36.740	0.170	18.385	0.078	104.886	0.006
28	*E. coli*	CTX-M9	28	BD2	CTX-M9(gad)_BD2_28cfu						
29	*E. coli*	CTX-M9	3	BD2	CTX-M9(gad)_BD2_3cfu						
30	*E. coli*	CTX-M9	0	BD2	CTX-M9(gad)_BD2_0cfu						
31	*E. coli*	BG_IPC	2828	BD1	IPC_gad_BD1_2828cfu	34.825	0.318	18.630	0.141	129.560	0.005
32	*E. coli*	BG_IPC	283	BD1	IPC_gad_BD1_283cfu	34.580	0.354	13.765	0.219	51.395	0.012
33	*E. coli*	BG_IPC	28	BD1	IPC_gad_BD1_28cfu	35.715	0.078	12.660	0.113	170.778	0.004
34	*E. coli*	BG_IPC	3	BD1	IPC_gad_BD1_3cfu	34.880	0.750	13.600	0.085	36.068	0.018
35	*E. coli*	BG_IPC	0	BD1	IPC_gad_BD1_0cfu	35.805	0.771	12.400	1.047	18.214	0.035
36	*E. coli*	BG_IPC	2828	BD2	IPC_gad_BD2_2828cfu	33.540	0.085	19.315	0.021	316.111	0.002
37	*E. coli*	BG_IPC	283	BD2	IPC_gad_BD2_283cfu	35.320	1.018	12.530	0.198	26.500	0.024
38	*E. coli*	BG_IPC	28	BD2	IPC_gad_BD2_28cfu	34.400	0.368	12.070	0.127	63.800	0.010
39	*E. coli*	BG_IPC	3	BD2	IPC_gad_BD2_3cfu	35.480	0.184	13.000	0.481	107.048	0.006
40	*E. coli*	BG_IPC	0	BD2	IPC_gad_BD2_0cfu	36.615	1.138	13.615	0.191	34.328	0.019
41	*K. pneumoniae*	*khe*	3831	BD1	khe_BD1_3831cfu	36.720	1.584	18.145	0.007	16.659	0.038
42	*K. pneumoniae*	*khe*	383	BD1	khe_BD1_383cfu	41.540		20.335	0.007		
43	*K. pneumoniae*	*khe*	38	BD1	khe_BD1_38cfu						
44	*K. pneumoniae*	*khe*	4	BD1	khe_BD1_4cfu						
45	*K. pneumoniae*	*khe*	0	BD1	khe_BD1_0cfu						
46	*K. pneumoniae*	*khe*	3831	BD2	khe_BD2_3831cfu	37.585	0.346	16.255	0.007	85.320	0.007
47	*K. pneumoniae*	*khe*	383	BD2	khe_BD2_383cfu	39.540		20.305	0.021		
48	*K. pneumoniae*	*khe*	38	BD2	khe_BD2_38cfu						
49	*K. pneumoniae*	*khe*	4	BD2	khe_BD2_4cfu						
50	*K. pneumoniae*	*khe*	0	BD2	khe_BD2_0cfu						
51	*K. pneumoniae*	OXA48	3831	BD1	OXA48(khe)_BD1_3831cfu	35.825	0.233	19.365	0.134	235.143	0.003
52	*K. pneumoniae*	OXA48	383	BD1	OXA48(khe)_BD1_383cfu	36.830		20.240	0.127		
53	*K. pneumoniae*	OXA48	38	BD1	OXA48(khe)_BD1_38cfu			39.150	0.580		
54	*K. pneumoniae*	OXA48	4	BD1	OXA48(khe)_BD1_4cfu			40.320			
55	*K. pneumoniae*	OXA48	0	BD1	OXA48(khe)_BD1_0cfu						
56	*K. pneumoniae*	OXA48	3831	BD2	OXA48(khe)_BD2_3831cfu	34.960	0.693	20.055	0.021	31.379	0.020
57	*K. pneumoniae*	OXA48	383	BD2	OXA48(khe)_BD2_383cfu	36.135	0.361	20.155	0.247	199.750	0.003
58	*K. pneumoniae*	OXA48	38	BD2	OXA48(khe)_BD2_38cfu			24.315	0.262		
59	*K. pneumoniae*	OXA48	4	BD2	OXA48(khe)_BD2_4cfu			25.775	0.078		
60	*K. pneumoniae*	OXA48	0	BD2	OXA48(khe)_BD2_0cfu						
61	*K. pneumoniae*	CTX-M15	3831	BD1	CTX-M15(khe)_BD1_3831cfu	36.925	0.092	14.725	0.163	444.000	0.001
62	*K. pneumoniae*	CTX-M15	383	BD1	CTX-M15(khe)_BD1_383cfu	37.510		15.950	0.184		
63	*K. pneumoniae*	CTX-M15	38	BD1	CTX-M15(khe)_BD1_38cfu						
64	*K. pneumoniae*	CTX-M15	4	BD1	CTX-M15(khe)_BD1_4cfu						
65	*K. pneumoniae*	CTX-M15	0	BD1	CTX-M15(khe)_BD1_0cfu						
66	*K. pneumoniae*	CTX-M15	3831	BD2	CTX-M15(khe)_BD2_3831cfu	35.755	0.361	13.140	0.127	137.061	0.005
67	*K. pneumoniae*	CTX-M15	383	BD2	CTX-M15(khe)_BD2_383cfu	37.130	1.061	16.105	0.049	29.406	0.022
68	*K. pneumoniae*	CTX-M15	38	BD2	CTX-M15(khe)_BD2_38cfu						
69	*K. pneumoniae*	CTX-M15	4	BD2	CTX-M15(khe)_BD2_4cfu						
70	*K. pneumoniae*	CTX-M15	0	BD2	CTX-M15(khe)_BD2_0cfu						
71	*K. pneumoniae*	BG_IPC	2828	BD1	IPC_khe_BD1_2828cfu	34.260	0.099	13.330	0.014	348.833	0.002
72	*K. pneumoniae*	BG_IPC	283	BD1	IPC_khe_BD1_283cfu	34.550	0.467	12.860	0.198	46.149	0.014
73	*K. pneumoniae*	BG_IPC	28	BD1	IPC_khe_BD1_28cfu	35.320	0.382	12.755	0.177	155.621	0.004
74	*K. pneumoniae*	BG_IPC	3	BD1	IPC_khe_BD1_3cfu	34.410	1.640	11.890	0.042	19.929	0.032
75	*K. pneumoniae*	BG_IPC	0	BD1	IPC_khe_BD1_0cfu	34.685	0.460	12.475	0.247	44.420	0.014
76	*K. pneumoniae*	BG_IPC	2828	BD2	IPC_khe_BD2_2828cfu	34.440	0.141	13.285	0.049	325.462	0.002
77	*K. pneumoniae*	BG_IPC	283	BD2	IPC_khe_BD2_283cfu	33.430	0.113	13.555	0.445	50.316	0.013
78	*K. pneumoniae*	BG_IPC	28	BD2	IPC_khe_BD2_28cfu	34.890	0.424	12.185	0.035	82.564	0.008
79	*K. pneumoniae*	BG_IPC	3	BD2	IPC_khe_BD2_3cfu	35.460	1.131	13.190	0.014	27.494	0.023
80	*K. pneumoniae*	BG_IPC	0	BD2	IPC_khe_BD2_0cfu	35.515	0.601	19.740	0.156	29.486	0.022
81	*E. faecium*	*aac6*	0	BD1	aac6_BD1_0cfu						
82	*E. faecium*	*aac6*	1	BD1	aac6_BD1_1cfu			13.905	0.049		
83	*E. faecium*	*aac6*	8	BD1	aac6_BD1_8cfu	37.880		16.455	0.078		
84	*E. faecium*	*aac6*	78	BD1	aac6_BD1_78cfu	37.530		13.840	0.028		
85	*E. faecium*	*aac6*	784	BD1	aac6_BD1_784cfu	35.880	0.919	11.150	0.014	38.641	0.016
86	*E. faecium*	*aac6*	0	BD2	aac6_BD2_0cfu						
87	*E. faecium*	*aac6*	1	BD2	aac6_BD2_1cfu						
88	*E. faecium*	*aac6*	8	BD2	aac6_BD2_8cfu						
89	*E. faecium*	*aac6*	78	BD2	aac6_BD2_78cfu			14.065	0.078		
90	*E. faecium*	*aac6*	784	BD2	aac6_BD2_784cfu	35.905	0.516	11.225	0.148	52.511	0.012
91	*E. faecium*	*vanB*	0	BD1	vanB_BD1_0cfu						
92	*E. faecium*	*vanB*	1	BD1	vanB_BD1_1cfu	38.020		14.605	0.092		
93	*E. faecium*	*vanB*	8	BD1	vanB_BD1_8cfu			15.035	0.064		
94	*E. faecium*	*vanB*	78	BD1	vanB_BD1_78cfu	38.430		13.420	0.028		
95	*E. faecium*	*vanB*	784	BD1	vanB_BD1_784cfu	36.455	0.106	10.240	0.014	308.412	0.002
96	*E. faecium*	*vanB*	0	BD2	vanB_BD2_0cfu						
97	*E. faecium*	*vanB*	1	BD2	vanB_BD2_1cfu			14.900	0.042		
98	*E. faecium*	*vanB*	8	BD2	vanB_BD2_8cfu						
99	*E. faecium*	*vanB*	78	BD2	vanB_BD2_78cfu			12.580	0.099		
100	*E. faecium*	*vanB*	784	BD2	vanB_BD2_784cfu	37.090	0.481	10.675	0.007	78.851	0.008
101	*E. faecium*	BG_IPC	0	BD1	IPC_aac6_BD1_0cfu	36.180	0.537	9.765	0.092	59.360	0.011
102	*E. faecium*	BG_IPC	1	BD1	IPC_aac6_BD1_1cfu	34.800	0.537	8.860	0.000	68.263	0.009
103	*E. faecium*	BG_IPC	8	BD1	IPC_aac6_BD1_8cfu	34.920	0.453	8.130	0.156	127.571	0.005
104	*E. faecium*	BG_IPC	78	BD1	IPC_aac6_BD1_78cfu	36.370	0.467	9.755	0.064	70.973	0.009
105	*E. faecium*	BG_IPC	784	BD1	IPC_aac6_BD1_784cfu	35.845	0.742	9.810	0.127	59.851	0.011
106	*E. faecium*	BG_IPC	0	BD2	IPC_aac6_BD2_0cfu	34.535	0.757	8.695	0.106	42.361	0.015
107	*E. faecium*	BG_IPC	1	BD2	IPC_aac6_BD2_1cfu	34.655	0.827	7.865	0.007	46.190	0.014
108	*E. faecium*	BG_IPC	8	BD2	IPC_aac6_BD2_8cfu	36.280	0.042	10.920	0.042	422.667	0.002
109	*E. faecium*	BG_IPC	78	BD2	IPC_aac6_BD2_78cfu	35.005	0.361	9.265	0.007	102.960	0.006
110	*E. faecium*	BG_IPC	784	BD2	IPC_aac6_BD2_784cfu	35.660		10.840	0.099		
111	*S. aureus*	*gapA*	1388	BD1	gapA_BD1_1388cfu	37.745	1.322	15.380	0.042	24.713	0.026
112	*S. aureus*	*gapA*	138	BD1	gapA_BD1_138cfu			17.710	0.042		
113	*S. aureus*	*gapA*	14	BD1	gapA_BD1_14cfu			21.470	0.014		
114	*S. aureus*	*gapA*	1	BD1	gapA_BD1_1cfu			41.365	0.148		
115	*S. aureus*	*gapA*	0	BD1	gapA_BD1_0cfu						
116	*S. aureus*	*gapA*	1388	BD2	gapA_BD2_1388cfu	35.600	0.127	29.040	0.014	65.600	0.010
117	*S. aureus*	*gapA*	138	BD2	gapA_BD2_138cfu	37.140		17.635	0.106		
118	*S. aureus*	*gapA*	14	BD2	gapA_BD2_14cfu						
119	*S. aureus*	*gapA*	1	BD2	gapA_BD2_1cfu			39.025	0.078		
120	*S. aureus*	*gapA*	0	BD2	gapA_BD2_0cfu						
121	*S. aureus*	*mecA*	1388	BD1	mecA(gapA)_BD1_1388cfu	37.745	1.322	20.270	0.113	20.439	0.031
122	*S. aureus*	*mecA*	138	BD1	mecA(gapA)_BD1_138cfu			23.910	0.269		
123	*S. aureus*	*mecA*	14	BD1	mecA(gapA)_BD1_14cfu						
124	*S. aureus*	*mecA*	1	BD1	mecA(gapA)_BD1_1cfu						
125	*S. aureus*	*mecA*	0	BD1	mecA(gapA)_BD1_0cfu						
126	*S. aureus*	*mecA*	1388	BD2	mecA(gapA)_BD2_1388cfu	35.600	0.127	27.740	0.071	56.143	0.011
127	*S. aureus*	*mecA*	138	BD2	mecA(gapA)_BD2_138cfu	37.140		22.475	0.049		
128	*S. aureus*	*mecA*	14	BD2	mecA(gapA)_BD2_14cfu						
129	*S. aureus*	*mecA*	1	BD2	mecA(gapA)_BD2_1cfu						
130	*S. aureus*	*mecA*	0	BD2	mecA(gapA)_BD2_0cfu						
131	*S. aureus*	BG_IPC	1	BD1	IPC_gapA_BD1_1cfu	37.470		10.675	0.148		
132	*S. aureus*	BG_IPC	784	BD1	IPC_gapA_BD1_784cfu			14.235	0.233		
133	*S. aureus*	BG_IPC	78	BD1	IPC_gapA_BD1_78cfu	35.880		12.460	0.099		
134	*S. aureus*	BG_IPC	8	BD1	IPC_gapA_BD1_8cfu	36.470		10.435	0.064		
135	*S. aureus*	BG_IPC	0	BD1	IPC_gapA_BD1_0cfu	33.790	0.141	8.715	0.092	716.429	0.001
136	*S. aureus*	BG_IPC	1	BD2	IPC_gapA_BD2_1cfu			13.100	0.156		
137	*S. aureus*	BG_IPC	784	BD2	IPC_gapA_BD2_784cfu	35.205	0.955	24.085	0.035	17.108	0.037
138	*S. aureus*	BG_IPC	78	BD2	IPC_gapA_BD2_78cfu	37.330		15.190	0.113		
139	*S. aureus*	BG_IPC	8	BD2	IPC_gapA_BD2_8cfu	37.445	0.021	13.425	0.021	800.667	0.001
140	*S. aureus*	BG_IPC	0	BD2	IPC_gapA_BD2_0cfu	37.145	0.445	12.115	0.148	119.190	0.005
